# Management of a Complex Fibroadenoma During Pregnancy

**DOI:** 10.7759/cureus.87289

**Published:** 2025-07-04

**Authors:** Mackenzie Wills, Karen Clary, Joel Yellin, Kelly Krupa

**Affiliations:** 1 Department of Breast Surgery, Lake Erie College of Osteopathic Medicine, Rochester, USA; 2 Department of Pathology, Rochester Regional Health, Rochester, USA; 3 Department of Breast Surgery, Rochester Regional Health, Rochester, USA

**Keywords:** breast surgery, complex fibroadenoma, core needle biopsy, fibroadenoma with cystic changes, lactating adenoma, lactational changes in pregnancy, milk-fistula formation, obstetrics, pregnancy

## Abstract

This case report describes a 25-year-old woman who presented with a complex fibroadenoma with multifocal cysts and lactational changes during pregnancy. The fibroadenoma grew rapidly during the patient’s pregnancy, necessitating surgical intervention postpartum. While conservative management is generally preferred during pregnancy and lactation, this case highlights the importance of prompt diagnosis, timely surgical intervention, and the management of diagnostic and surgical challenges in pregnancy.

## Introduction

Fibroadenomas are the most common benign breast tumor in young women and can be classified into small, giant, simple, or complex types [1]. Giant fibroadenomas, which are hormone-sensitive, can grow rapidly during pregnancy, requiring careful management. While conservative management is often the first approach, surgical intervention may be necessary if there is a significant increase in size that can result in breast deformities or significant diagnostic uncertainty due to the potential risk of malignancy [2]. This case report details a fibroadenoma that grew rapidly during pregnancy, requiring diagnostic core biopsy intrapartum and surgical excision postpartum.

## Case presentation

A 25-year-old woman presented with a rapidly enlarging mass in her left breast six days after childbirth. She reported initially noticing a lump before pregnancy, but it had significantly grown by the third month of pregnancy, increasing even more postpartum. This enlargement resulted in difficulties expressing milk from the left breast, and consequently, the patient primarily breastfed from the unaffected right breast.

**Figure 1:**
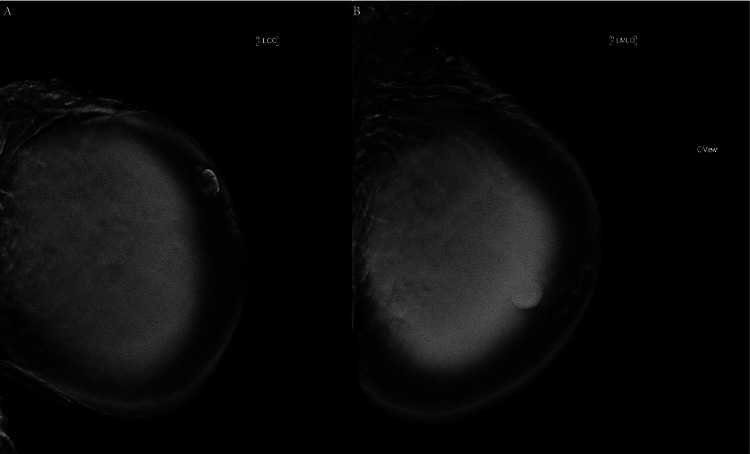
(A) Left cranio-caudal (LCC) and (B) left mediolateral oblique (LMLO) mammography views of the breast mass.

On physical examination, the left breast was roughly double the size of the right, with a large mass in the upper portion. The skin over the mass was thin, erythematous, hyperpigmented, with visible superficial veins. Her previous imaging was reviewed, and her ultrasound revealed a large, complex mass occupying most of the left breast, with multiple fluid-filled spaces. Her mammogram showed dense tissue with a hyperdense, well-defined mass measuring up to 18 cm, suggestive of a lactating adenoma. At the time of this imaging, a biopsy was discussed and postponed due to the risk of milk fistula formation. However, after further discussion with the patient regarding the risk of milk fistula formation versus the risk of diagnostic uncertainty and potential for malignancy, a core needle biopsy was performed approximately two weeks later. This biopsy revealed a fibroepithelial lesion with sclerosing adenosis, cysts, hemorrhage, and necrosis, consistent with a complex fibroadenoma. Follow-up imaging, including ultrasound and mammogram, was performed and confirmed a solid-cystic mass measuring 10.9 x 11.8 x 14.2 cm with increased vascularity. Left breast diagnostic mammogram images are shown in Figure 1. Surgical removal was then recommended.

The patient consulted a plastic surgeon to discuss her concerns about breast asymmetry after mass removal. A mastopexy was discussed as a possible future option depending on the postoperative cosmetic outcome. Due to the size of the mass and risk of a possible malignant phyllodes tumor, which accounts for 2.5% of all fibroepithelial lesions in women of European descent, with higher rates in Asian women, the patient underwent surgical excision one month after her initial evaluation with the breast surgery group [3].

**Figure 2:**
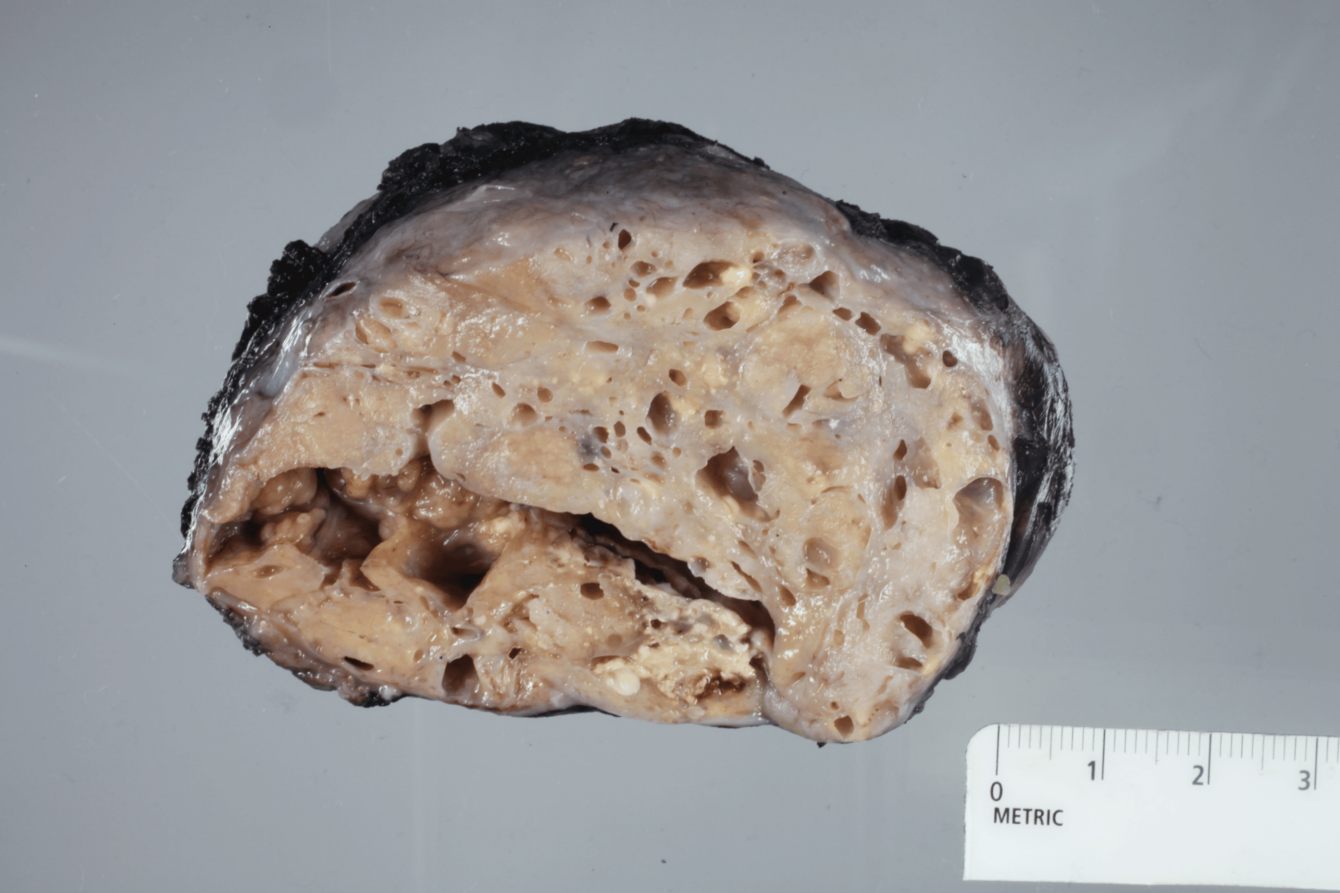
Gross pathology specimen measuring 11.7 x 10.5 x 5.7 cm showing a well-circumscribed mass with a deep crypt, numerous cystic changes, and focal solid areas.

Preoperatively, the possibility of aspirating cystic fluid to reduce the size of the mass was considered but ultimately deemed unnecessary, as several pockets had already decreased in size before the operation. A 9 cm incision was made along the inframammary fold to optimize cosmetic results and preserve options for future plastic surgery. During the surgical procedure, the posterior aspect of the mass was inadvertently opened with cautery, resulting in milk drainage, which was managed by placing sutures to close the defect. The mass, measuring 12 cm x 8 cm x 3 cm, was then carefully excised. The surgical site was irrigated, and hemostasis was achieved before applying a hemostatic agent to the cavity. Due to the mass effect, redundant skin remained in the upper inner quadrant and was not excised to preserve future cosmetic options. The deep dermis and skin were closed, and a sterile gauze dressing was applied. The mass was sent for final pathology. The gross pathology image is shown in Figure 2.

**Figure 3:**
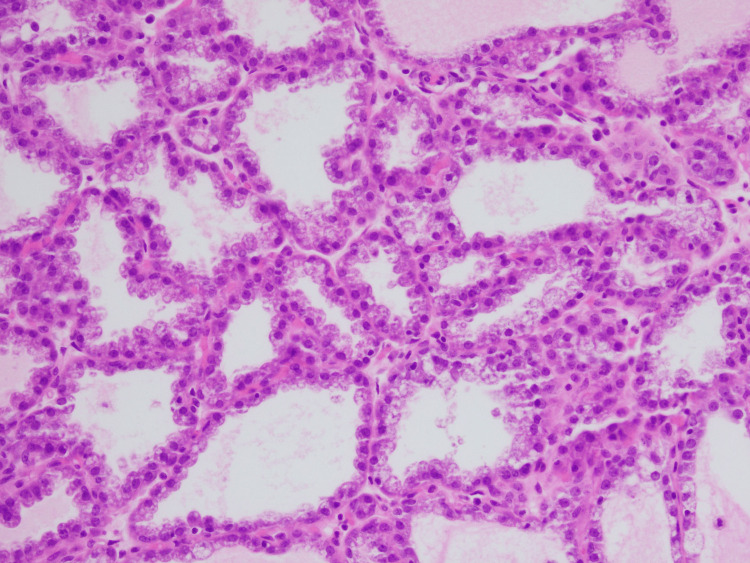
A 20X magnification image showing cuboidal cells with abundant vacuolated cytoplasm and focal eosinophilic secretions indicating lactational changes.

**Figure 4:**
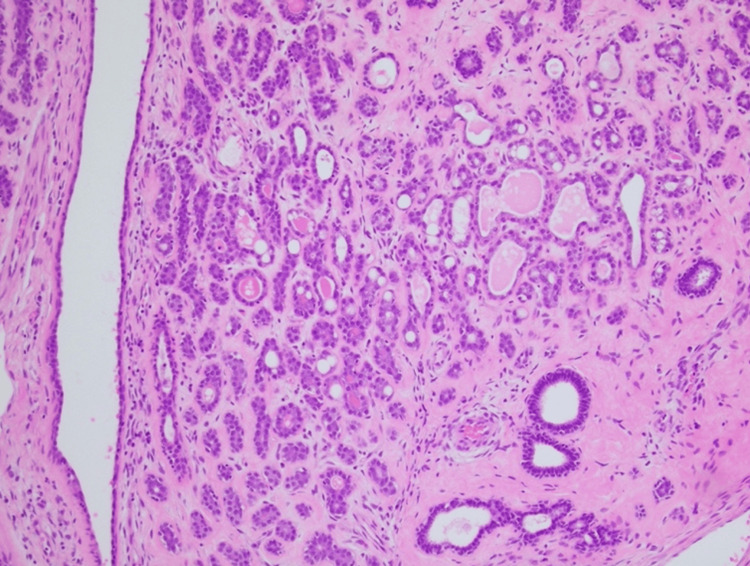
A 10X magnification image showing a nodule of adenosis comprised of small benign ductules and stromal fibrosis.

**Figure 5:**
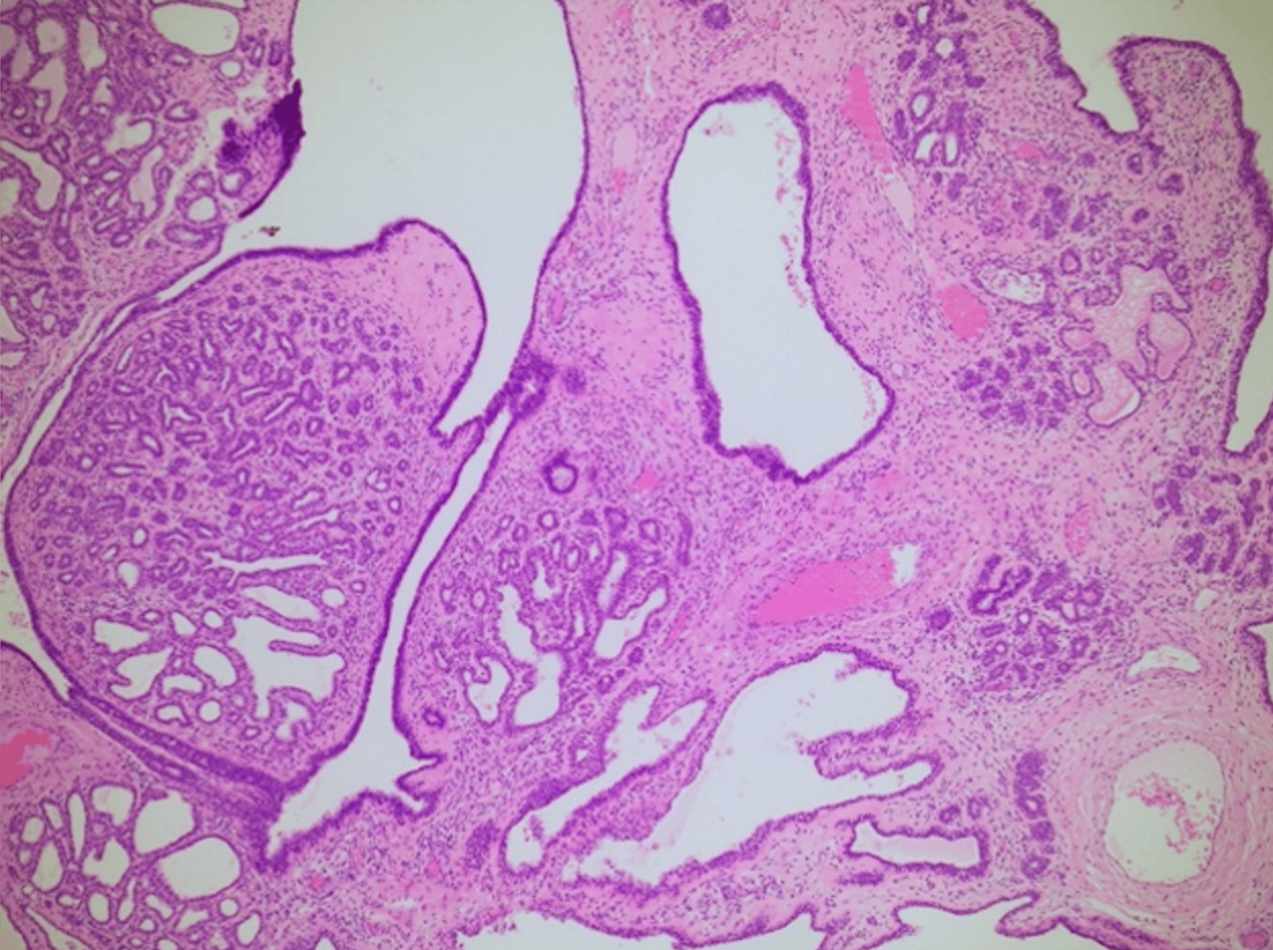
A 4X magnification image showing significant complex cystic formation with adenosis in a lobulocentric pattern.

Histopathological analysis confirmed the presence of a complex fibroadenoma with multifocal cysts and lactational changes. Histopathology images are shown in Figures 3-5. 

Postoperative ultrasound was performed and showed no abnormalities, and since the patient was pleased with the cosmetic result, she opted against further plastic surgery.

## Discussion

Fibroadenomas are considered the most common benign neoplasm of the breast and are frequently seen in pregnancy. They are a subtype of fibroepithelial lesions and typically grow slowly, measuring less than 3 cm in young women aged between 20 and 35 years, although this can vary. Fibroadenomas are generally mobile, rubbery, painless lesions and may occur bilaterally in multiple locations or unilaterally. They account for 68% of all breast masses, with an incidence of 2.2% in adolescent populations, and have various descriptors that can further classify fibroadenomas [4].

Giant fibroadenomas may show a focally prominent intracanalicular pattern, which can mimic the leaf-like pattern of phyllodes tumors. Although similar, the benign giant fibroadenoma lacks the stromal atypia and mitotic activity of a phyllodes tumor. Lactating adenomas, another type of fibroadenoma, may develop during pregnancy and lactation. The architecture of a lactating adenoma shows varying degrees of lactational changes due to physiological states of pregnancy. These adenomas can infarct, which should signify a need to rule out a mimicking carcinoma [5]. Cystic fibroadenomas are fibroadenomas that undergo cystic changes. They are particularly rare and carry an increased risk of developing breast carcinoma. Current guidelines from the National Comprehensive Cancer Network recommend that fine needle aspiration (FNA) provides an effective way to evaluate cyst composition [6]. Ultimately, the current management of fibroadenomas is variable and consists of observation, imaging, core needle biopsy, and potentially surgical excision to rule out malignancy [7].

Therefore, performing a complete evaluation of a pregnant patient with a lump should always be pursued with reassurance that potential complications such as radiation damage or milk fistula formation are extremely rare. The dose of a four-view mammogram is 0.4 mrad without any protection of the abdomen. With additional shielding such as protective lead drapes, radiation exposure is considered nonsignificant and safe for fetal growth and maturity [8]. In addition, core biopsy should be strongly advised to help establish a definitive diagnosis and to rule out malignancy [9]. According to a recent retrospective cohort study, the risk of milk fistulas after core biopsies is low, and indicated procedures should be pursued, not modified or avoided. Patients can be reassured that with proper management, such as avoiding massage and encouraging pumping/emptying as needed, a fistula formation can resolve within a few days to a week. On a rare occasion, if an incision is made very close to the nipple and becomes irritated, it may take longer to resolve. However, the risk-benefit ratio of not identifying a malignancy should be discussed and considered [10].

A handful of case reports discuss the management of fibroadenomas in pregnancy. In a case report of a 27-year-old pregnant patient, her giant fibroadenoma was surgically excised two months postpartum, indicating that surgical intervention can be performed without complication during lactation [11]. In another case report, a 26-year-old woman presented at 15 weeks pregnant with a unilateral mass of the right breast. After 12 weeks of observation and conservative management, she returned to the clinic due to significant pain and enlargement of the mass. A biopsy was repeated to rule out malignancy, and an urgent lumpectomy was scheduled due to concern for pain and significant deformity of the breast. The mass was excised under general anesthesia, and the condition of the 30-week-old fetus was monitored by cardiotocography before and after surgery. No harmful side effects to the fetus were observed, and the patient recovered well without any complications. This unique case also highlighted the importance of surgical intervention even during pregnancy to prevent breast tissue damage [1]. Similarly, another case report describes a 17-year-old female patient who was 24 weeks pregnant and diagnosed with a giant fibroadenoma in the right breast. Due to significant pain and deformity of her right breast, a biopsy was performed to confirm the diagnosis, and excision was performed under general anesthesia. Fetal heart rate and activity were monitored, and an incision at the inframammary fold was performed for potential plastic reconstruction in the future. The patient’s pregnancy continued uncomplicated, and she decided to opt out of plastic surgery [12].

Although women of childbearing age presenting with breast masses are most likely to have benign conditions, breast cancer is one of the most common malignancies that complicate pregnancy. The index of suspicion for cancer must be high for women with a breast mass presenting in the gestational or lactational period. The physiological changes of pregnancy could mask symptoms due to breast enlargement. This, paired with low suspicion among providers, could potentially result in late diagnosis and late-stage disease [13]. One case report describes a 37-year-old female patient who originally presented with a breast lump in pregnancy and was preliminarily diagnosed with fibroadenoma with cystic changes. However, the patient deferred biopsy until after pregnancy and decided to pursue observation management. Pregnancy was uneventful until 39 weeks, when she was diagnosed with intrauterine fetal demise. She opted to excise the breast mass under general anesthesia. Final surgical pathology of the mass revealed grade 3 invasive ductal carcinoma present upon histopathological examination [14]. This case highlights the importance of discussing with patients the risk of diagnostic uncertainty and risk of malignancy and encourages prompt diagnosis with mammography and core needle biopsy, with subsequent surgical excision, if necessary, even in pregnancy.

## Conclusions

This case report highlights a rare presentation of a rapidly enlarging fibroadenoma during pregnancy, which poses significant diagnostic and management challenges. Despite the potential risks associated with surgical intervention during lactation, such as cosmetic deformity, mindful incision placement and open discussion about plastic surgery options can be done to prevent structural damage. Ultimately, this case report demonstrates the need for careful monitoring and timely intervention in managing complex fibroadenomas during pregnancy, particularly when rapid growth occurs, to establish a definitive diagnosis, relieve symptoms, and prevent further deformity to the breast.
